# Oxidative stress and ion channels in neurodegenerative diseases

**DOI:** 10.3389/fphys.2024.1320086

**Published:** 2024-01-29

**Authors:** Razan Orfali, Adnan Z. Alwatban, Rawan S. Orfali, Liz Lau, Noble Chea, Abdullah M. Alotaibi, Young-Woo Nam, Miao Zhang

**Affiliations:** ^1^ Neuroscience Research Department, Research Centre, King Fahad Medical City, Riyadh, Saudi Arabia; ^2^ BrainExperiments.com, Montreal, QC, Canada; ^3^ Department of Biomedical and Pharmaceutical Sciences, Chapman University School of Pharmacy, Irvine, CA, United States

**Keywords:** antioxidants, calcium channel, neurodegenerative disorders, oxidative stress, potassium channels, reactive oxygen species, sodium channels, glutathione

## Abstract

Numerous neurodegenerative diseases result from altered ion channel function and mutations. The intracellular redox status can significantly alter the gating characteristics of ion channels. Abundant neurodegenerative diseases associated with oxidative stress have been documented, including Parkinson’s, Alzheimer’s, spinocerebellar ataxia, amyotrophic lateral sclerosis, and Huntington’s disease. Reactive oxygen and nitrogen species compounds trigger posttranslational alterations that target specific sites within the subunits responsible for channel assembly. These alterations include the adjustment of cysteine residues through redox reactions induced by reactive oxygen species (ROS), nitration, and S-nitrosylation assisted by nitric oxide of tyrosine residues through peroxynitrite. Several ion channels have been directly investigated for their functional responses to oxidizing agents and oxidative stress. This review primarily explores the relationship and potential links between oxidative stress and ion channels in neurodegenerative conditions, such as cerebellar ataxias and Parkinson’s disease. The potential correlation between oxidative stress and ion channels could hold promise for developing innovative therapies for common neurodegenerative diseases.

## 1 Introduction

**FIGURE 1:**
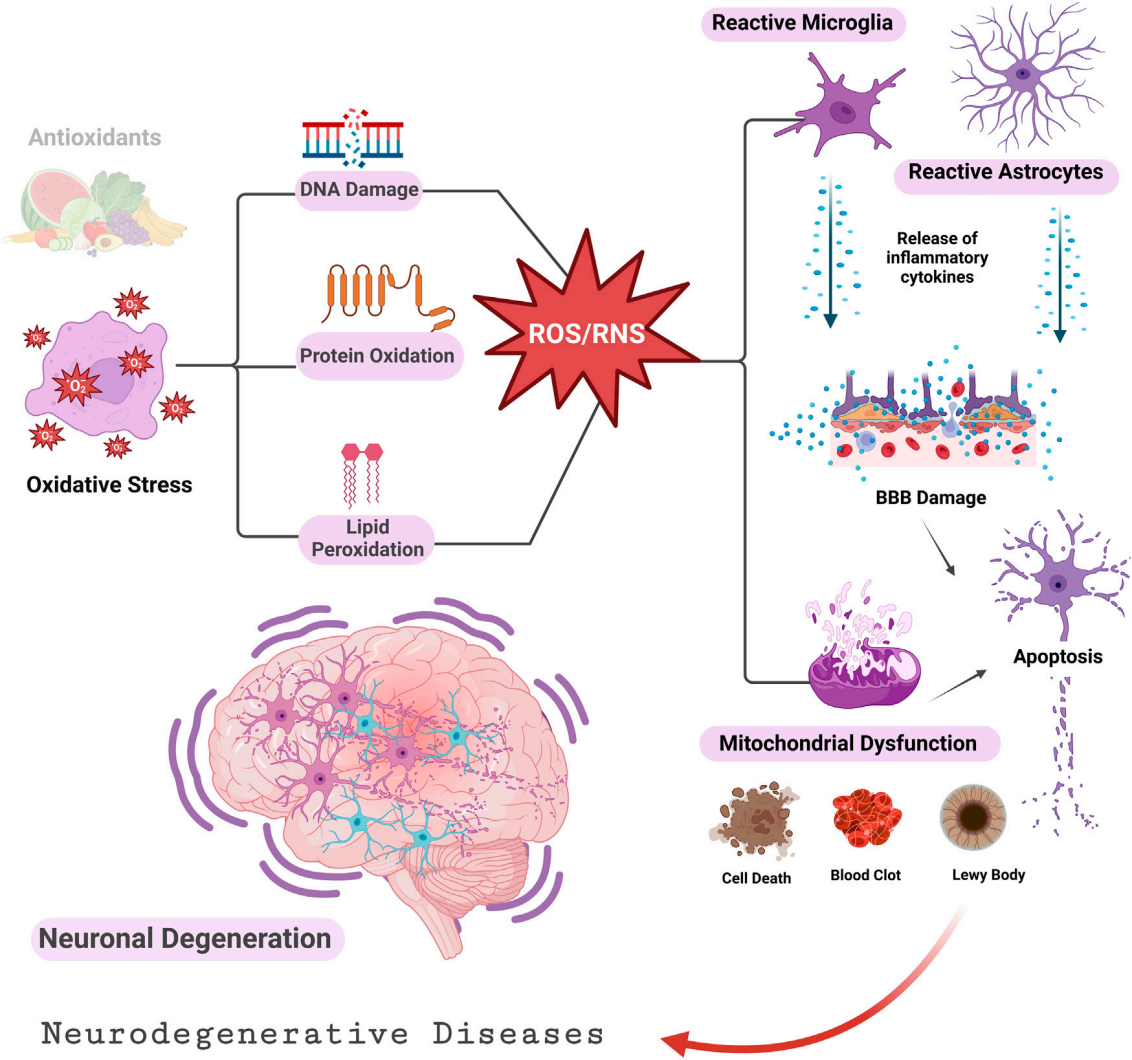
Oxidative stress and neurodegenerative diseases (Ashok et al., 2022). An imbalance between ROS/RNS and antioxidants damages lipids, proteins, and DNA. Cellular apoptosis and tissue death are promoted by impaired mitochondrial function and buildup of activated astrocytes and microglia. BBB, Blood Brain Barrier.

Reactive oxygen species (ROS) are generated by living organisms as a result of their regular cellular metabolic processes and environmental factors, such as smoking, air pollutants, UV radiation, alcohol consumption, infections, non-steroidal anti-inflammatory drugs (NSAIDs), and inflammation (Valko et al., 2006; Birben et al., 2012). ROS are required in small to moderate amounts for normal cellular functions. However, elevated concentrations induce detrimental alterations to proteins, DNA, and lipids, which hinder cell function (Liguori et al., 2018; Uttara et al., 2009). The lack of antioxidants generates oxidative stress that increases reactive species’ levels (Uttara et al., 2009). Pathological states often result in intracellular oxidative agents overtaking reducing agents, causing redox imbalances and oxidative stress (Simon et al., 2013; Sies et al., 2017). Numerous oxidative stress-related diseases have been reported (Sies et al., 2017; Ramírez et al., 2016), such as neurodegenerative disorders involving Parkinson’s (Henchcliffe and Beal, 2008), Alzheimer’s (Gella and Durany, 2009; Chang et al., 2014), spinocerebellar ataxia (Guevara-García et al., 2012), Huntington’s disease (Browne et al., 1999), and amyotrophic lateral sclerosis (ALS) (Cunha-Oliveira et al., 2020). Several cardiovascular diseases are also linked with oxidative stress, such as hypertension (Griendling et al., 2021), heart failure (Pagan et al., 2022), myocardial ischemia (Kurian et al., 2016), and atherosclerosis (Kattoor et al., 2017). Other pathologies linked to oxidative stress involve obesity (Manna and Jain, 2015), chronic inflammation (Orzechowski et al., 2019), and chronic pain (Kaushik et al., 2020). The literature is rich in presenting compelling evidence of a significant association between neurodegenerative disorders, aging, and oxidative stress (Browne et al., 1999; Gella and Durany, 2009; Uttara et al., 2009; Riverón et al., 2010; Chang et al., 2014; Cunha-Oliveira et al., 2020). Oxidative stress causes neuroinflammation, and mitochondrial dysfunction leads to apoptosis and cell damage that triggers neurodegenerative processes (Figure 1) (Selivanov et al., 2011; Ashok et al., 2022). Several neuroprotective therapies have been developed to combat ROS that damage neurons and cause neurodegenerative disorders (Uttara et al., 2009). The intracellular redox status can significantly alter the gating properties of ion channels (Akbarali, 2014). Indeed, various neurodegenerative diseases result from altered ion channel function and mutations (Li and Lester, 2001).

This review is an effort to summarize some of the common modifications in ion channel regulations by ROS in some neurodegenerative disease states.

## 2 Oxidative stress and ion channels

**FIGURE 2:**
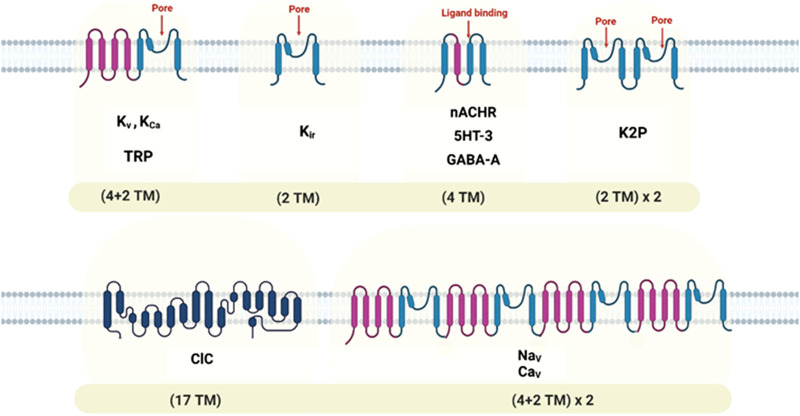
Topology diagrams of some ion channel families, showing locations of transmembrane domains and pore-forming segments. Transmembrane, (TM).

Oxidative stress passively damages proteins, lipids, and DNA but also directly modulates many molecules in the cell signaling network, such as ion channels. The ion channel is a macromolecular pore in cell membranes that selectively conducts Na+, K+, Ca2+, and Cl–ions. These pores are essential in conducting the ions across cell membranes (Litalien et al., 2011). Different stimuli open ion channels and conduct ions into or out of the cells, including changes in membrane potential, chemical stimuli, or mechanical deformation (Li and Lester, 2001). According to the stimulus they respond to, ion channels can be classified into three superfamilies: voltage-gated (Purves et al., 2001a), ligand-gated (Purves et al., 2001b), or mechanosensitive (Martinac, 2012). Ion channel subtypes are differentiated by their primary structure, distribution, and functional properties (Zheng and Trudeau, 2023). In voltage-gated ion channels, the membrane potential changes, and a specific ion is selectively dissolved; these channels can be categorized into different families based on the ion specificity (Purves et al., 2001a). Neurotransmitters or other ligands can trigger ligand-gated ion channels. There are several subtypes of ligand-gated channels, just like voltage-gated channels. Mechanosensitive ion channels respond to alterations in mechanical forces on the cell membrane (Zheng and Trudeau, 2023). Ion channels play a fundamental role in nerve conduction, neural communication, and muscle contraction, in addition to their conical function of transporting ions across the cell membrane to set membrane potential (Rosendo-Pineda et al., 2020) (Table 1) (Figure 2). Detailed information on recent ion channel types can be found in the excellent textbook by Zheng and Trudeau et al. (Zheng and Trudeau, 2023). There are also multiple reviews on specific types of ion channels (Purves et al., 2001b; Shah and Aizenman, 2014; de Lera Ruiz and Kraus, 2015; Nam et al., 2023a).

**TABLE 1:** A Summary of different ion channel types and their role in the CNS (Zheng and Trudeau, 2023).

Family	Subtype/Subfamily	Subunit/Topology transmembrane (TM)	Role in the CNS	References
Voltage-gated Ca^2+^ channels (Ca_v_)	Ca_v_ 2.1 (N- Type)	(4 + 2 TM) x 4	Release of neurotransmitters and Ca^2+^ ion transients within dendrites	Purves et al. (2001a), Catterall (2011), Schampel and Kuerten (2017)
	Ca_v_ 2.2 (P/Q -Type)			
	Ca_v_ 2.3(R - Type)			
	Ca_v_ 3. (1–3)(T-Type)		Frequent firing and pace-making	
Voltage-gated Na^+^ channels (Na_v_)	Na_V_1.1, Na_V_1.2, Na_V_1.3 and Na_V_1.6	(4 + 2 TM) x 4	Action potential initiation, transmission, and modulation of neuronal circuits	de Lera Ruiz and Kraus (2015), Wang et al. (2017), Barbieri et al. (2023), Zheng and Trudeau (2023)
Voltage-gated K^+^ channels (K_v_)	K_V_1 - K_V_4	(4 + 2 TM)	Mediate outward K^+^ currents. Setting the resting potential and repolarizing action potentials (limit neuronal excitability)	Purves et al. (2001a), Shah and Aizenman (2014), Zheng and Trudeau (2023)
	K_V_7 (KCNQ)			
	Eag (K_V_10- K_V_12)			
Other related K^+^ channels	Ca^2+^ activated K^+^ channels	(4 + 2 TM)	Control cellular excitability and maintain K^+^ homeostasis in non-excitable cells	WEAVER et al. (2006), Aldrich et al. (2021), Orfali and Albanyan (2023), Orfali et al. (2023), Rahman et al. (2023)
	BK (K_Ca_1.1)			
	SK (K_Ca_2)—voltage independent			
	Two-Pore domain K^+^ channels (K_2_P- Leaky K^+^ channels): TWIK, TREK, TASK, TALK, THIK, and TRESK	(2 TM) x 2	Maintain the stability of the resting membrane conductance and contribute to the repolarization of action potentials in excitable cells	Aggarwal et al. (2021), Fan et al. (2022)
	Inwardly rectifying K^+^ channels: K_ir1–7_	(2 TM)	Control of cellular excitability and K^+^ ion homeostasis	Butt and Kalsi (2006), Adelman et al. (2023)
Other cation-channels	Transient Receptor Potential Channels (TRP channels): TRPC, TRPM, and TRPV	(4 + 2 TM)	Neuronal firing and synapse transmission	Sawamura et al. (2017), Wang et al. (2020), Lee et al. (2021)
	Hyperpolarization-Activated Cyclic Nucleotide-Gated channels: HCN_1-4_	(4 + 2 TM)	Play a key role in modulating synaptic transmission, dendritic integration, and neuronal excitability	Shah (2014), DiFrancesco and DiFrancesco (2015), Chang et al. (2019)
Voltage-gated Cl^−^ channels (ClC)	ClC-1	(17 TM)	Set the cell resting membrane potential and maintain proper cell volume	Rinke et al. (2010), de Lera Ruiz and Kraus (2015), Shen et al. (2021)
	CIC-2	The membrane does not span all helices		
Ligand-gated channels	Nicotinic acetylcholine receptor (nACHR)	(4 TM)	Fast synaptic transmission in the nervous system and at the neuromuscular junction	Purves et al. (2001b), Li et al. (2014), Zheng and Trudeau (2023)
	Serotonin receptor (5HT-3)	(4 TM)	Fast synaptic transmission	
	GABAA receptor (GABA-A)	(4 TM)	Fast inhibitory transmission	
Mechanosensitive ion channels	Piezo (Piezo1)	(38 TM)	Regulation of neural growth and development, neuroinflammation, and angiogenesis	Martinac (2012), Jin et al. (2020), Harraz et al. (2022)

Posttranslational oxidative modifications of certain proteins, such as ion channels, can result from imbalances in cellular redox state caused by ROS production, ineffective antioxidant defenses, or environmental oxidative stress. (Bogeski and Niemeyer, 2014; Kiselyov and Muallem, 2016). In ion channels and other proteins, cysteine residues are the most vulnerable to oxidation due to their highly reactive thiol groups. It is possible to oxidize thiols into sulfonic acids and sulfonic based on the amount of oxidant present, the redox potential, the amount of charge, and the temperature. Various oxidative modifications can be applied to cysteines, including processes like glutathionylation and nitrosylation. Elevated levels of ROS can lead to the decomposition of amino acids, such as lysine and arginine, into aldehydes or the conversion of methionine residues into sulfoxides and sulfones (Bogeski and Niemeyer, 2014). Reactive nitrogen species (RNS) and ROS have the potential to directly alter ion channels by nitrosylation, nitration, and oxidation of specific amino acid residues. This can eventually affect the signaling pathways that modulate channel function, modifying gene transcription, turnover, proteasomal degradation, and trafficking (Akbarali, 2014). Sensitivity to alterations in the side chains of the amino acid residues that serve as the targets for ROS/RNS is typically associated with the presence of sulfur atoms in (methionine and cysteine), aromatic rings (tryptophan, histidine, and phenylalanine), or hydroxyl groups (tyrosine), (Annunziato et al., 2002; Akbarali, 2014; Miranda et al., 2023). In biological systems, there is a physiological balance between the generation of ROS and their detoxification through antioxidant scavengers, such as glutathione, catalase, and superoxide dismutase. When there is an imbalance, oxidative stress occurs (Gulcin, 2020).

**FIGURE 3:**
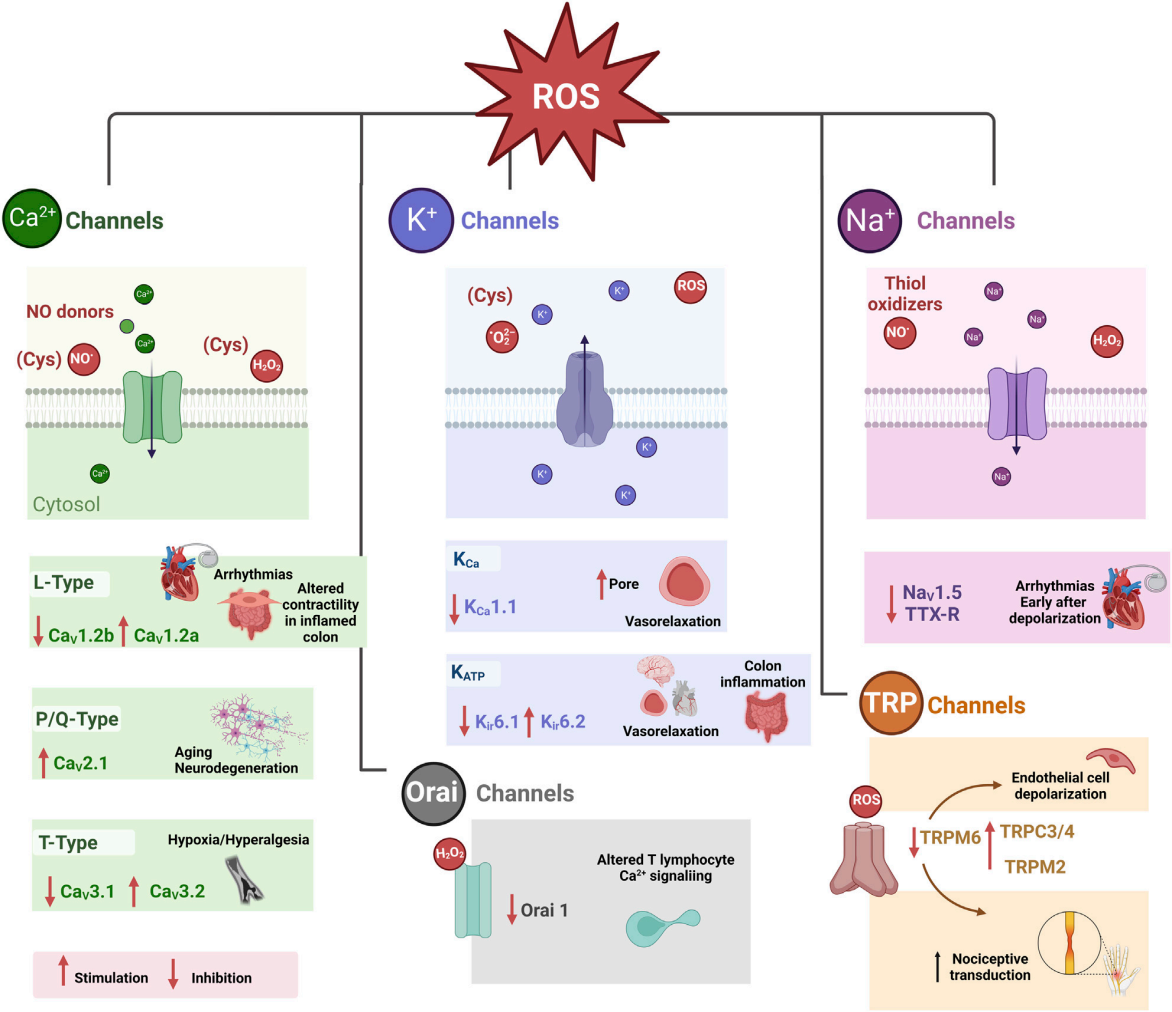
Pathophysiological consequences of redox modulation of some ion channels (Akbarali, 2014). Cysteine (CyS), Store-operated Ca^2+^ release-activated Ca^2+^ (CRAC) channels or (Orai 1 channels). Tetrodotoxin-resistant (TTX-R) Na^+^ (Na_V_1.9) TTX-R.

Many types of ion channels are recognized to be modulated by oxidative stress. This modulation can be beneficial in some, while in others, it leads to pathological states (Figure 3) (Akbarali, 2014). For example, alterations in the gating properties and ion selectivity of voltage-gated ion channels may occur. Oxidative stress can also affect ligand-gated ion channels, thereby altering their signaling pathways and sensitivity (Miranda et al., 2023). Another consequence of oxidative stress on regulating intracellular Ca^2+^ levels is its ability to alter the function of Ca^2+^ release channels within the endoplasmic reticulum and Ca^2+^ uptake channels in the cell’s plasma membrane (Santulli et al., 2017). Ion channels might also function as sensors of redox changes, given that various ion channels are closely linked to oxidative stress. Also, ROS-induced damage can be restored through natural protective mechanisms. Consequently, for therapeutic purposes, understanding how these reactants affect ion channel functionality is essential to understanding how oxidative stress-related diseases are triggered.

### 2.1 Regulation of ion channels by antioxidants

Post-translational modifications (PTMs) are significant mechanisms modulating the functions of ion channels. Protein phosphorylation is a classical PTM, and protein kinases regulate many ion channels throughout phosphorylation (Yang et al., 2014). There are different types of PTMs, such as Ubiquitylation, S-glutathionylation, O-glycosylation, etc. Both normal and abnormal states, including oxidative stress, are linked to post-translational modifications (PTMs) mediated by redox processes targeting cysteine residues’ thiol group. Redox-mediated post-translational modifications (PTMs) constitute a significant set of PTMs that specifically target the thiol group of cysteine residues. These modifications are evident in various physiological and pathological contexts, including situations marked by oxidative stress. Redox-mediated post-translational modifications (PTMs) constitute a significant group of PTMs that specifically affect the thiol group of cysteine residues. These modifications are evident in various physiological and pathological conditions characterized by oxidative stress (Moran et al., 2001; Dalle-Donne et al., 2008; Yang et al., 2014). One prominent mechanism for redox-mediated thiol modulation is S-glutathionylation, which involves adding a glutathione (GSH) group to the protein. The presence of reactive oxygen species (ROS) plays a vital role in facilitating S-glutathionylation. This phenomenon is increasingly observed in various ion channels, including voltage-gated Ca^2+^ channels and ATP-sensitive K^+^ channels (K_ATP_) (Tang et al., 2011; Yang et al., 2011).

Reduced GSH is a significant non-enzymatic antioxidant in mammalian cells (Averill-Bates, 2023). GSH is a tripeptide composed of glycine, cysteine, glutamate, and the active thiol group in the cysteine residue that acts as a potent antioxidant. This antioxidant GSH is produced within the cell’s cytoplasm and transported to the mitochondria (Wadey et al., 2009). Hydrogen sulfide (H_2_S) has long been regarded as toxic, but it is now being found to play an important physiological role (at low concentrations). Nitric oxide (NO) is another well-known gaseous intracellular signal transducer, including H_2_S. H_2_S is produced from cysteine by several enzymes and plays a physiological role in cell signaling regulation, homeostasis, and combating oxidative species, such as ROS/RNS, in the body (Olas, 2015; Shefa et al., 2018).

Studies on the modulation of ion channel functions by NO and H_2_S are summarized in Table 2.

**TABLE 2:** A summary of studies on NO, H_2_S modulation affecting ion channels. Nitric oxide (NO), Hydrogen sulfide (H_2_S).

Channel	Modulator	Effect	References
K_V1-6_	NO	Block, suppress	Brock et al. (2001), Núñez et al. (2006), Spiers and Steinert (2021)
K_ATP_	H_2_S	H_2_S-activated K_ATP_ channels	Tang et al. (2010)
	NO	NO-activated K_ATP_ channels	Kawano et al. (2009), Spiers and Steinert (2021)
K_Ca_	H_2_S	H_2_S-activated small and Intermediate conductance K_Ca_ channels	Tang et al. (2010)
	NO	NO-suppressed SK currents	Klyachko et al. (2001), Dalle-Donne et al. (2008), Artinian et al. (2012), Kyle et al. (2013), Spiers and Steinert (2021)
		NO-increased BK current	
Ca_V_	H_2_S	H_2_S-inhibited L-type Ca^2+^ channels in cardiomyocytes	Tang et al. (2010)
	NO	H_2_S-stimulated the same channels in neurons	Almanza et al. (2007), Chen et al. (2002), D’Ascenzo et al. (2002)
		NO-activated L- and P/Q-type, whereas R and N-type channels are unaffected	
Na_V_	NO	Reduction of Na_V_ currents	Scheiblich and Steinert (2021), Spiers and Steinert (2021)
TRP	H_2_S	H_2_S-activated TRPV1 &TRPA1	Tang et al. (2010)
ClC	H_2_S	Activated Cl^−^ channel	Tang et al. (2010)

### 2.2 Ion channel mutations and oxidative stress

Most known human ion channel diseases or channelopathies are hereditary and investigated through genetic approaches (Li and Lester, 2001). Genetic analysis studies can be challenging because the clinical phenotypes are complex, and significant genetic heterogeneity exists. In other words, mutations in different genes may lead to the same clinical phenotype. Despite these challenges, numerous genes associated with human diseases have been successfully identified, characterized, and localized by applying molecular genetic techniques (Li and Lester, 2001; Nam et al., 2023a). Genome-wide association studies (GWAS) have linked ion channels to oxidative stress-related disorders (Akbarali, 2014; Ramírez et al., 2016; Liguori et al., 2018). The consequences of these ion channel mutations related to oxidative stress are diverse and contribute to the pathogenesis of various diseases, such as neurological disorders, cardiac arrhythmias, and certain types of cancers. Moreover, the aging process is linked with increased oxidative stress and a higher incidence of ion channel dysfunctions (Uttara et al., 2009; Chang et al., 2014; Kurian et al., 2016; Liguori et al., 2018). Understanding the relationship between ion channel mutations and oxidative stress is essential for developing targeted therapeutic strategies. Detailed information on specific potassium channel mutations and oxidative stress-related disorders, such as ataxias, can be found in these excellent manuscripts (Figueroa et al., 2010; Duarri et al., 2012; Duarri et al., 2012; Lee et al., 2012; Nam et al., 2023a). This review will generally cover different ion channel mutations caused by oxidative stress in neurodegenerative diseases.

## 3 Oxidative stress in neurodegenerative disorders

Neurodegenerative disorders affect millions of people worldwide. Brain atrophy is the hallmark of neurodegenerative diseases due to constant decline in neuronal function. Despite age being a significant risk factor for all neurodegenerative disorders, recent research indicates that genetic makeup and environmental factors greatly influence the risk as well (Chen et al., 2012; Lamptey et al., 2022). Although neurodegenerative disorders have distinct etiologies and develop in different brain sites, recent studies have observed that their effects on cellular and molecular mechanisms are similar (Aborode et al., 2022; Gates et al., 2022; Teleanu et al., 2022; Rehman et al., 2023). The central nervous system (CNS) has a significant oxidative potential because of its elevated oxygen usage. However, the CNS is particularly vulnerable to oxidative stress because of the abundance of readily oxidizable substances, limited levels of primary and secondary antioxidants, elevated iron content in specific brain regions, the generation of ROS by various internal mechanisms, and the presence of non-replicating neurons (Maher, 2006; Adibhatla and Hatcher, 2010; Guevara-García et al., 2012). Figure 1 demonstrates the tendency of neurodegenerative diseases to progress as a result of oxidative stress (Teleanu et al., 2022). Cells malfunction and even undergo apoptosis because the redox balance shifts to oxidative (Lew et al., 2022). Various neurodegenerative disorders are believed to be impacted by oxidative stress (Figure 1). Ion channels’ dysregulation is another common pathophysiologic mechanism that causes degenerative CNS diseases of widely differing genetic etiologies (Huang and Shakkottai, 2023). Furthermore, H_2_S at low concentrations lowers the level of ROS and thus protects neurons from oxidative stresses (Shefa et al., 2018). The inhaled form of H_2_S has a neuroprotective role In a Parkinson’s disease mouse model (Kida et al., 2011). It also protects neurons from apoptosis and degeneration (Olas, 2015).

Oxidative stress has been suggested as a factor in the development of various neurodegenerative disorders, including certain types of ataxias. The etiology of the diseases is multifaceted, with genetic and familial investigations underscoring their heterogeneity (Guevara-García et al., 2012). Point mutations often lead to diminished expression of proteins specific to the mutated genes. The connection between neurodegenerative disorders and oxidative stress is dependent on molecular, *in vitro*, and animal studies findings. Nonetheless, conflicting results emerge from human biomarker studies, indicating the necessity for additional research on the role of redox in neurodegenerative disorders associated with channelopathies (Li and Lester, 2001; Guevara-García et al., 2012).

Listed below are some findings that correlate with the modulation of ion channels and overproduction of ROS with neurodegenerative disorders, such as inherited cerebellar ataxia and Parkinson’s disease.

### 3.1 Inherited cerebellar ataxia

Inherited Cerebellar Ataxias (ICAs) combine a group of complex and uncommon neurodegenerative conditions that impact the cerebellum, spinal cord, and peripheral nerves (Coarelli et al., 2023). A person with ICA can experience balance, gait, speech, limb movement, eye movement, and cognitive difficulties. A significant correlation exists between ataxia location and cerebellar neuropathology: hemisphere lesions result in limb or appendicular ataxia, while midline lesions result in gait ataxia (Kashyap et al., 2020). Spinocerebellar ataxia (SCA) is a subgroup of hereditary cerebellar ataxia, a progressive, neurodegenerative, heterogeneous, rare disease that affects the cerebellum (Brooker et al., 2021; Bhandari et al., 2023). The pathology of spinocerebellar ataxia is still unknown, but the principal cells involved in degeneration are Purkinje cells (Koeppen, 2005). Purkinje cells regulate fine movement and muscle coordination. Thus, a decline in the normal firing of the Purkinje cells leads to an excessive calcium influx and excitotoxicity (Koeppen, 2005; Hosy et al., 2011). In the CNS, particularly the cerebellum, histopathology shows atrophy and enlargement of the lateral ventricles, loss of myelin in the frontal horn of the spinal cord, and axonal degeneration (Bhandari et al., 2023).

#### 3.1.1 ICA and oxidative stress

There is an association between oxidative stress and several neurological disorders, including hereditary ataxias (Guevara-García et al., 2012; Lew et al., 2022). Numerous investigations have been conducted to prove the therapeutic roles of antioxidants in ICAs (Sarva and Shanker, 2014; Braga Neto et al., 2016; Picher-Martel and Dupre, 2018). Nevertheless, the results indicated that these antioxidants only partially alleviated symptoms of ICAs. This limitation may be because of the emphasis on clinical outcomes rather than a comprehensive understanding of the underlying molecular mechanisms associated with their approach to addressing oxidative stress (Picher-Martel and Dupre, 2018; Lew et al., 2022). The cause of ICAs is diverse (Coarelli et al., 2023). The link between ataxia and oxidative stress depends mainly on molecular, *in vitro*, and *in vivo* studies. Recent findings, for example, have indicated that ataxin 2 and others are linked with the redox imbalance in this disease (Guevara-García et al., 2012).

The significance of understanding the influence of oxidative stress on ion channels is crucial in considering ataxias. It is also needed to develop innovative approaches via alternative therapeutic intervention in ICA and related diseases.

#### 3.1.2 Ion channels involved in oxidative stress-related ataxia

A cerebellar cortex includes Purkinje cells that integrate all input into the cerebellum (Hosy et al., 2011; Hirano, 2018; Huang and Shakkottai, 2023). A common feature of cerebellar ataxia is cerebellar atrophy and Purkinje neuron degeneration (Koeppen, 2005; Cocozza et al., 2021). Purkinje neurons are unique in that they spike independently of synaptic stimulation. SCA mouse models demonstrate that disruptions in the firing in Purkinje neurons considerably weaken motor function, indicating that this pacemaking ability of Purkinje neurons plays a critical role in motor coordination (Kurian et al., 2016; Bhandari et al., 2023; Dell’Orco et al., 2015; Jayabal et al., 2016). In resting conditions, Purkinje neurons fire at an average frequency of 40 Hz with unvarying inter-spike interval duration. Ion channels are predominantly responsible for maintaining this regularity (Raman and Bean, 1999; Braga Neto et al., 2016; Huang and Shakkottai, 2023).

In Purkinje neurons, voltage-gated Na^+^ channels (Na_v_1.6 and Na_v_1.1) initiate action potentials when they are activated. Then, the voltage-gated K^+^ channel activation will mediate the repolarization of the action potential. Ca^2+^ enters Purkinje neurons via voltage-gated Ca^2+^ channels, mainly Ca_v_2.1 and Ca_v_3.1, upon depolarization. When Ca^2+^ binds to a specific type of K^+^ channel, called Ca^2+^-activated K^+^ channels, an outward K^+^ current is generated, and the Purkinje neuron is hyperpolarized to produce the afterhyperpolarization (AHP) (Cooper and Jan, 1999; Li and Lester, 2001; Burke and Bender, 2019). The main Ca^2+^-activated K^+^ channels that generate the AHP in Purkinje neurons are small-conductance Ca^2+^-activated K^+^ channels type 2 (K_Ca_2.2) channels (Kasumu et al., 2012; Nam et al., 2017; Nam et al., 2023b) and large-conductance Ca^2+^-activated K^+^ (BK) channels (Du et al., 2020) (Table 3).

**TABLE 3:** Summary of some ion channels involved in oxidative stress-related neurodegenerative disorders.

Channel	Subtype	Function	Neurodegenerative diseases	References
Ca_V_	Ca_V_ 2.1 (P/Q-type)	Inward Ca^2+^ current upon depolarization	SCA*6 Episodic ataxia type 2	Zhuchenko et al. (1997), Bushart and Shakkottai (2019)
	Ca_V_3.1 (P/Q-type)		SCA42 PD	Morino et al. (2015)
				Tabata et al. (2018)
Na_V_	Na_V_1.1	Na^+^ ion influx and membrane depolarization during the action potential	PD	Saunders et al. (2016), Wang et al. (2019)
	Na_V_1.1			
K_V_	K_V_3.3	K^+^ influx when the membrane depolarizes, leading to hyperpolarization	SCA13	Figueroa et al. (2010), Zhang and Kaczmarek (2016)
	K_V_4.3		SCA19 and SCA22	Duarri et al. (2012), Lee et al. (2012)
K_Ca_	K_Ca_1.1	Outward K^+^ current upon activation, fast AHP** in neurons	SCA	Staisch et al. (2016), Du et al. (2020)
	K_Ca_2.2	Medium AHP in neurons	Cerebellar ataxia, SCA2	Klockgether et al. (2019), Nam et al. (2023b), Rahman et al. (2023)
			PD	Lam et al. (2013)
TRP	TRPC3	Mediate neuronal differentiation and vasomotor function, inhibit the release of cytokines and NO.	SCA41	Fogel et al. (2015)
			PD	Rather et al. (2023)
	TRPV4	Mediates inflammation pathways	PD	Vaidya and Sharma (2020), Liu et al. (2022)
*Spinocerebellar Ataxia (SCA)				
**Afterhyperpolarization (AHP)				

Because ion channels play an important function in Purkinje neuron physiology, maintaining redox equilibrium is crucial to maintaining neurons’ homeostasis due to the alteration of their activity by oxidative stress. Enzymatic antioxidants, such as superoxide dismutase, glutathione peroxidases, and catalase, along with non-enzymatic antioxidants like GSH and vitamins A, C, and E, counteract various types of oxidative stress (Irato and Santovito, 2021). These antioxidants, whether endogenous or exogenous, reduce oxidative stress and scavenge ROS in ICAs, which could pave the way for a new ICA treatment (Pandolfo, 2008; Lew et al., 2022). Activating antioxidative transcription factor NRF2 could be a viable strategy to alleviate oxidative damage in ICAs (Kavian et al., 2018). In response to oxidative stress, NRF2 modulates key antioxidant enzymes, which, either directly or indirectly, regulate redox homeostasis (Liu et al., 2016; Lew et al., 2022). AM-36 is a neuroprotective agent that combines antioxidant and Na^+^ channel blockade properties (Callaway et al., 2001). Compared to agents possessing only one of these actions, AM-36 inhibited toxicity and apoptosis (mediated by the generation of ROS) (Callaway et al., 2001). Therefore, it is important to understand the link between the strategy targeting specific ion channels and antioxidants in mediating the progression of ICAs.

### 3.2 Parkinson’s disease

Parkinson’s disease (PD) is a common neurodegenerative condition distinguished by the progressive degeneration of dopaminergic cells in the substantia nigra, a region in the midbrain known for its accumulation of synuclein (Poewe et al., 2017). The dopamine secretion by these neurons is crucial for controlling movement ease and balance. Multiple pathways and mechanisms are involved in the underlying molecular pathogenesis, including synthase proteostasis, oxidative stress, mitochondrial function, neuroinflammation, Ca^2+^ homeostasis, and axonal transport (Miraglia et al., 2015; Poewe et al., 2017). The etiologies of PD are still questionable. Leucine-rich repeat kinase 2 (LRRK2) Mutations are one of the causative genetic variants that account for several autosomal, dominantly inherited PD (Blauwendraat et al., 2020). It has also been discovered that other genes, including ATP13A2, SNCA, PINK, GIGYF2, HTRA2, and DJ1, can cause familiar and early-onset PD. Among their functions are the degradation of ubiquitin proteins, the response to oxidative stress, apoptosis, cell survival, and mitochondrial function (Maiti et al., 2017).

#### 3.2.1 Parkinson’s diseases and oxidative stress

Oxidative stress significantly promotes the erosion of dopaminergic neurons in PD (Dias et al., 2013). Oxygen is essential for brain function, and a large amount of oxygen is converted into ROS. Overproduction of ROS in the brain raises oxidative stress in people with Parkinson’s disease (Chang and Chen, 2020). Oxidative stress is closely related to other components of the degenerative process, like excitotoxicity, nitric oxide toxicity, and mitochondrial dysfunction (Jenner, 2003; Henchcliffe and Beal, 2008). Several genes associated with familial PD, including parkin, alpha-synuclein, LRRK2, DJ-1, and PINK-1, have been identified, providing important understandings of the molecular pathways underlying the disease pathogenesis, as well as highlighting earlier mysterious mechanisms where oxidative stress plays a role in the disease (Dias et al., 2013). Examination of brain tissue in Parkinson’s disease patients reveals a reduced level of GSH compared to glutathione disulfide (GSSG) compared to healthy brain tissue (Sian et al., 1994; Pearce et al., 1997). As oxidative stress leads to programmed cell death, the mitochondrial condition of GSH has gained recognition as a significant indicator of this occurrence (Chang and Chen, 2020).

#### 3.2.2 Ion channels involved in oxidative stress-related Parkinson’s

Targeting ion channels provides an intriguing mechanistic strategy to address the progression of PD and other neurodegenerative disorders because of their important roles in neuronal activities (Braga Neto et al., 2016). K^+^ channels are important in neuronal excitability, neurotransmitter release, neuroinflammation, and synaptic transmission in PD pathology. In dopaminergic neurons, voltage-dependent K^+^ currents mediate repolarizing action potentials and fine-tune pacemaker firing rates (Braga Neto et al., 2016; Picher-Martel and Dupre, 2018). Voltage-gated Na^+^ channels modulate pacemaker frequency (Chen et al., 2012; Sarva and Shanker, 2014).

Tetrodotoxin inhibits the voltage-gated Na^+^ channels, revealing slow oscillatory potentials regulated by L-Type voltage-gated Ca^2+^ channels that regulate the precision, frequency, and robustness of peacemaking (Zaichick et al., 2017) (Table 3). While excessive firing and excess Ca^2+^ are significantly studied in connection to neurodegeneration, the direct function of the related ion channels in PD pathogenesis is mysterious. A variety of Parkinson’s disease animal models, ranging from toxin-induced to genetically modified mice, exhibit abnormalities in the operation of different ion channels (Daniel et al., 2021). The pathogenesis of Parkinson’s disease encompasses numerous interconnected pathways, including protein aggregation, oxidative stress, mitochondrial impairments, and abnormalities in autophagy. As a result, there have been numerous efforts to address these pathways in order to provide neuroprotection (Daniel et al., 2021). While several of these drugs in preclinical studies have demonstrated positive outcomes, none of these interventions have effectively transitioned into clinical application (Jenner, 2003).

In the field of ion channel drug discovery, a significant challenge is preventing side effects arising from both target and off-target mechanisms. Additionally, subtype selectivity is challenging when various homologous members belong to the same subfamily (Brown et al., 2020; Chen et al., 2023).

## 4 Concluding remarks

Ion channel malfunction is a common factor in neurological disorders, even when various genes are implicated as the root causes of these diseases. The malfunction of ion channels can result from changes in the intracellular redox environment, which alter how these channels function. Consequently, oxidative stress shows a significant role in the onset and development of neurodegenerative conditions involving ataxias, Parkinson’s, Alzheimer’s, and ALS. Despite recent advancements, the precise mechanisms of reactive oxygen species (ROS)-mediated neurodegenerative diseases remain partially understood. The role of ion channels in neurodegenerative disorders associated with oxidative stress has now been recognized, as they experience functional adjustments in such conditions. However, the significance of targeting ion channels therapeutically varies depending on the disease and the tissues in which these channels are active. Ultimately, neurodegenerative diseases may be effectively treated with a combination of ion channel-modulating therapy and antioxidant medication. More research on the function of ion channels in oxidative stress may provide a platform for exploring new therapeutic approaches for treating many neurodegenerative diseases associated with oxidative stress.

## Abbreviations

(ALS), Amyotrophic lateral sclerosis; (AHP), Afterhyperpolarization; (Ca^2+^), Calcium; (CNS), Central nervous system; (GWAS), Genome-wide association studies; (GSH), Glutathione; (H2S), Hydrogen sulfide; (ICAs), Inherited Cerebellar Ataxias; (BK), Large-conductance Ca^+2^-activated K^+^; (LRRK2), Leucine-rich repeat kinase 2; (LTCC), L-type voltage-gated Ca2+channel; (NO), Nitric oxide; (NSAIDs), Non-steroidal anti-inflammatory drugs; (nACHR), Nicotinic acetylcholine receptor; (PD), Parkinson’s disease; (PTMs), Post-translational modifications; (K^+^), Potassium; (ROS), Reactive oxygen species; (RNS), Reactive nitrogen species; (K_Ca_2.x or SK), Small-conductance Ca^2+^-activated K^+^; (TMs), Transmembrane helices; (K_2_P), Two-Pore domain K^+^ channels; (TRP), Transient Receptor Potential; (SCA), Spinocerebellar ataxias; (K_V_), Voltage-gated K^+^; (Na_V_), Voltage-gated Na^+^; (Ca_V_), Voltage-gated Ca^2+^.
